# Differences in impact of long term caregiving for mentally ill older adults on the daily life of informal caregivers: a qualitative study

**DOI:** 10.1186/1471-244X-13-103

**Published:** 2013-03-27

**Authors:** Marian I Zegwaard, Marja J Aartsen, Mieke HF Grypdonck, Pim Cuijpers

**Affiliations:** 1 Department of Psychiatry of the Elderly, Altrecht Mental Health Care, Oude Arnhemseweg 260, Zeist 3705 BK, the Netherlands; 2 Faculty of Social Sciences, VU-University Amsterdam the Netherlands, Amsterdam, the Netherlands; 3 Nursing Science, Ghent University, Ghent, Belgium; 4 Clinical Psychology, VU-University Amsterdam, Amsterdam, the Netherlands

**Keywords:** Older adults, Mental illness, Informal caregiver, Gain, Loss, Psychiatric nursing

## Abstract

**Background:**

Owing to the policy of extramuralization of care in most Western countries older people with severe mental illness have to rely more and more on informal caregivers for daily care. Caregivers themselves are often aged, and although caregiving implies an impact on daily life that exceeds the boundaries of usual informal care, the impact differs across caregivers. Some caregivers seem to suffer more than others, and the differences cannot be fully understood by factors currently known to exacerbate the burden of caregiving. In order to help caregivers reduce the impact of caregiving it is important to gain a deeper understanding of factors influencing the burden and its impact on the caregiver’s life. With this in mind, the aim of the study is to explore and understand differences in the impact of long-term caregiving on the quality of life of caregivers who look after older adults with severe mental illness.

**Methods:**

A qualitative, associative, inductive strategy and continuous simultaneous coding were used to interpret the data of 19 semi-structured interviews.

**Results:**

We identified an underlying psychological factor “perceived freedom of choice” which explains the gross differences in impact, leading to a definition of two main types of caregivers. Depending on how people perceive freedom of choice to provide care, the consequences of caregiving can be characterized as a process of gain (type 1) or loss (type 2). Four influential factors deepen the impact of caregiving for the type 2 caregivers, and two subtypes are identified for this category. Consequences of caregiving are most readily seen in a deteriorating quality of the relationship with the care recipient and in the psychosocial well-being of the caregiver.

**Conclusions:**

The concept of freedom of choice adds to our understanding of the differences and explains the variation in impact on the caregivers’ life. The type 1 caregiver generally experiences gain whereas type 2 generally experiences loss, which puts the latter group typically at risk of becoming overloaded. Whether people perceive that they have freedom of choice in caregiving is an important consideration in evaluating the type of intervention needed to support caregivers.

## Background

In recent decades, the policy of extramuralization of care in most Western countries has led to an increased number of older people with severe mental illnesses living in the community. Hence, these older people with severe mental illnesses (hereafter referred to as *care receivers*) have to rely increasingly on informal caregivers (hereafter *caregivers*) for their support in daily living [1,2]. Severe mental illness such as schizophrenia, bipolar disorder, depression and anxiety disorders can have a serious impact on the daily life of sufferers and their caregivers. The caregiver is confronted with long-term care for a person who sometimes inhabits a phenomenological world that is inaccessible and incomprehensible to healthy people. These care receivers often cannot conform to usual rules of social settings, may engage in inimitable behaviour and sometimes deny that they are ill. The mental illness often has a progressive course and is frequently accompanied by a high prevalence of acute and chronic somatic illnesses, with adverse effects of medication influencing the symptoms of the mental illness and increasing the risk of relapse.

Many, often aged, caregivers become involved in long-term caregiving which may interfere with numerous aspects of their daily life and exceed the boundaries of usual informal care. They invest a significant amount of time and energy in long-term caregiving, involving tasks that may be unpleasant [3]. Altogether, this increases the risk of becoming overloaded [3-7] which can severely impair quality of life and potentially lead to withdrawal from the caregiving situation. Therefore caring for these caregivers is an important issue in community care.

For community care to be effective there is a strong need for support interventions tailored to the individual situation. Such interventions require knowledge and insight concerning the processes and factors that help to understand the impact of caregiving on daily life. So far, research has come up with several stress-process oriented models suggesting that perceived burden must be understood through the individual appraisal of stressors and the availability and use of internal and external resources that buffer the negative effects of stressors on mental and physical health [8-11]. Studies in recent decades describe determinants that might contribute to the emergence of perceived burden [3,5,7,12-20]. Some studies focus on the caregivers’ emotional responses to the illness of the care receiver, such as anger, grief, and feelings of hopelessness [21-24]. Despite their important contributions to the understanding of the concept of perceived burden, these studies only partially explain why some caregivers seem to suffer more than others. The differences cannot be fully understood by factors currently known to exacerbate the burden of caregiving. This qualitative study seeks to explore and understand underlying factors that may shed new light on the caregivers' appraisal of the situation, which may cause these differences in impact.

## Method

A qualitative study was conducted to clarify and interpret caregivers’ experiences and perceptions as well as the processes underlying long-term caregiving.

### Procedure

Community mental health care nurses from two large Dutch mental health care organizations in the Netherlands invited caregivers to participate in the study. They explained the purpose of the study to the care receivers and their caregivers. If the caregiver was willing to participate, written information was given and an informed consent was signed. Those who agreed to participate were approached by the first author and arrangements for an interview were made. Recruitment took place from July 2007 to November 2008. The study was approved by the ethics committee of Altrecht Mental Health Care.

### Participants

Caregivers were eligible for inclusion when they spoke Dutch, were the most important caregiver (as judged by the nurse), had been a caregiver for at least 6 months, were caring for a person aged at least 60 years who had severe functional psychiatric illness - and problematic behaviour (from the caregiver’s perspective). The care receiver had to be community dwelling, but may have been temporarily admitted to a psychiatric hospital due to a crisis.

For ethical reasons, caregivers were not approached if the nurse judged that the interview might cause too much grief or anxiety in either the caregiver or the care receiver, or when the relationship between the caregiver and the care receiver was too severely disturbed.

Twenty-four caregivers were approached for the study (see Procedure). Five refused to participate, because they did not want to be reminded of the many years of sadness and uncertainty they as caregivers had gone through. None of the caregivers who agreed to participate subsequently withdrew from the study. Table 1 shows the demographic and background information of the participating caregivers.

**Table 1:** Demographic and background information of caregivers (N=19)


Age in years	M=66, SD=9.85
Type of relationship with care receiver	
Partner	12 (male 6)
Child	3 (male 2)
Friend or Relative	4 (male 2)
Self reported health problems of caregivers	
Physical problems	11
Depression	1
Duration of care (years)	M=24, Range=2-40

The mean age of the caregivers was 66, ranging from 48 to 77 for men and from 51 to 82 for women. Eleven caregivers reported having disabling conditions, such as fibromyalgia, diabetes or high blood pressure. One caregiver reported medication treatment for depression. All spouses and one child shared the household with the care-receiver, often interrupted by prolonged hospital admissions. Caregivers' socio-economic background ranged from lower to upper middle SES.

### Data collection

The caregivers were interviewed during one face-to-face interview. The interviews were audio-taped and lasted between 60 and 90 minutes. A topic list (see Additional file 1) based on literature to situations that might influence the caregiving burden provided direction to the interviews. Caregivers were asked to describe details of events, situations and conversations with the care-receiver, related to caregiving in their daily life.

To give the caregiver the opportunity to speak as freely as possible during the interview, it was sought to create a pleasant atmosphere. For this reason the interviews were conducted at a location suggested by the caregiver (mostly their own home), and in the care receiver's absence.

The interviews followed the natural course of conversation. The topic list was used to introduce those topics that were not introduced spontaneously by the interviewee. Questions were asked to get in to more detail about what was brought in by the interviewee. Field notes concerning impressions gained during the interview and information given after the tape recorder was turned off were noted immediately afterwards. This procedure generated sensitive and personal interview material on the impact of caregiving on the daily life of the respondents.

### Data analysis

The analyses were conducted in a cyclical process in which coding and thinking theoretically were used alternately [25]. A research team of three members (M.I.Z., M.J.A., M.G.), including both interviewers (M.I.Z, M.J.A.) was involved in the entire process of data analysis through the final results. As a first step, two researchers (M.I.Z. and M.G.) each read five transcripts in full to acquire an overall picture of the situation. Analytical thoughts and ideas with respect to the data were discussed in order to reach an understanding of the respondents’ point of view [26]. Notes were made about the first concepts pertinent to the interviews [27]. To refine the emerging theory, further interviews were conducted, and the established concepts and themes were alternate confronted with the input of new material.

During meetings M.I.Z. and M.G. constantly compared their interpretations of the data and worked towards consensus about the interpretation of possible meanings. Commonalities, differences, and explanations for differences between interviews were discussed for a more thorough understanding of the caregivers’ perspective and experiences. Comparing and contrasting elements within and between cases enabled disclosure of what was shared and what was different. A reflection on this analysis was described, text parts were coded and a code tree was developed. Coding was supported by the software program MAXqda.

For the purposes of improved researcher triangulation, a third researcher (M.J.A.) was involved in the analysis. She critically questioned the conclusions based on the interpretation of the data. This process provides an external check on the research. During these meetings all three worked together in checking the interpretation of the data against existing data and new materials. As such we constantly verified whether interpretations corresponded to the original interviews. New codes were added and the code-tree was restructured in accordance with theoretical insights. Coding and concept description were conducted simultaneously, facilitating the interpretative analytical process that best relates to the experience of the caregivers. Concepts were further categorized and main themes emerged [27,28]. Relations between categories and between themes were established and categories developed.

## Results

“Perceived freedom of choice” explains the gross differences in impact, distinguishing two types of caregiver: those who perceive caregiving as a voluntary act of compassion (type 1) and those who find caregiving to be an unavoidable obligation (type 2). Type 1 caregivers generally perceive caregiving as a process of gain; type 2 caregivers as a process of loss. The impact of freedom of choice is most visible in the quality of the relationship and the caregiver's psychosocial wellbeing. In the following section, first a description of “freedom of choice” is given. Next, differences in impact on the quality of the relationship and psychosocial wellbeing are described for the two types. We conclude with a discussion of four influential factors i.e., acceptance, home environment, feelings of competence and social relationships, that further subdivide the type 2 caregiver into two subtypes.

### Perceived freedom of choice

Perceived freedom of choice is defined as a non-conscious psychological state in which the caregiver feels he/she could choose to stop being a caregiver. This perceived freedom of choice is the underlying key concept which leads to two possible outcomes.

The caregivers who experience caregiving as voluntarily, contributing to a better life for the care receiver, base their support on sympathy or compassion. They are motivated by caregiving for its own sake. They do not provide all care. For them it is more important that caregiving is well organized. In this situation caregiving is considered as satisfying and enriching and they scarcely experience any feeling of burden.

For those who do not perceive freedom of choice, caregiving is seen as a logical consequence of their shared lives and its interconnectedness. Therefore, they feel that they are called on to undertake and provide for all daily matters in caregiving. Caregiving is, in their experience, unavoidable and inescapable. For these caregivers it is impossible to stop caregiving because this would be tantamount to abandoning the care-receiver (or: giving up the relationship). Under these conditions caregiving is leading to loss, grief or impoverishment.

### Domains in daily life

Caregiving mainly affects two domains of daily life: the quality of the relationship with the care-receiver and the caregiver's own psychosocial wellbeing. Aspects of relationship quality that may be affected include expectations, equality, togetherness, and respect. The psychosocial well-being of the caregiver is affected by the presence or absence of grief and mourning, autonomy and meaning, and participation in social life.

### Quality of the relationship

#### Expectations

All caregivers talk about changes in the quality of the relationship. The main difference between the two types of caregiver lies in the way they adapt their own expectations.

Type 1. Perceived freedom of choice. Caregivers who experience freedom of choice provide care in a loving and caring way. Because their lives are not interwoven with the care receiver's, acceptance of the illness and its consequences is easier, and they do not expect anything in return. They adapt their expectations to the limitations of the care receiver and this allows them to remain tolerant. In their view, the relationship is based on a tacit mutual commitment which is meaningful for both themselves and the care receiver.

Type 2. No perceived freedom of choice. For these caregivers the care-receiver’s illness and his/her behaviour constantly undermine their expectations about “mutuality” in their relationship and in the relationship with significant others. Over the years they find it continuously confirmed that it is impossible to share with the care receiver any of the household responsibilities or other obligations, or intimacy and mutuality in facing life's problems. They are faced with behaviour by the care receiver that does not correspond to generally accepted norms. Still, they feel others expect them to be in control of the situation or to take care of the consequences. These caregivers experience a lack of responsiveness on the part of the care receiver. As their lives are interwoven, they find it impossible to lower their expectations, making them oscillate between hope and disappointment. However, this does not keep them from trying to reach a desired mutual bond. As their efforts fail, for some caregivers grief turns into disappointment and frustration.

#### Equality

The behaviour of the care-receiver does not reflect the accepted norms and values within “normal” (social) relationships, and as a result the equality based on recognition and respect is disrupted.

Type 1 caregivers accept the inequality. They recognize that the older adult is often unable to contribute to a household, a job or social roles in life. Nonetheless, they strive for autonomy and normalcy. They refrain from taking over decision-making. With this attitude they try to strengthen the capabilities of the care-receiver*.*

Type 2. Most of the partners who share the household with the care receiver, and some of the children, experience an overwhelming “24/7 responsibility”. These caregivers hope for and even expect equality. However the round-the clock confrontation with the consequences of the illness and the person they feel responsible for is the immediate cause of disappointment in many interactions. Efforts to encourage the care-receiver to participate in household tasks often prove to be in vain. Due to uncertainty about what can be demanded given the mental illness and the often increased physical frailty, the caregiver is afraid to insist on participation. This leads to what caregivers see as an unavoidable and definitive loss of roles. The caregiver becomes more and more the main actor. According to caregivers “a kindly initiated dialogue” constantly turns into an “imposed decision”. Equality within the relationship is further disrupted by the absence of reciprocity from the care-receiver. Some type 2 caregivers are able to “interpret” reciprocity. For instance, one of the respondents regards her husband’s consent to being admitted to a nursing home every six weeks as an expression of appreciation for all that she as caregiver has to endure. Other type 2 caregivers felt that the disturbed behaviour can diminish the reciprocity between partners to such an extent that it is like “having a child rather than a partner”.

#### Togetherness

Togetherness is the sense of familiarity and belonging which is based on being able to tackle problems together. Most caregivers see moments of togetherness as an important feature of the quality of the relationship. In their view the care receiver’s ability to contribute to togetherness has changed due to the symptoms of the mental illness and the use of medication necessary to control the illness.

Type 1 caregivers actively seek togetherness. Their reward for their caregiving efforts is contained in the caregiving itself. Their support is merely that of an affectionate involvement, as in friendship. Throughout the many years of involvement during which they’ve gone through ups and downs, these caregivers experience a deepening of their relationship. They realize the damage the mental illness has caused in the lives of these older adults and they respect the way they handle this damage. There is respectful communication between them in which they suss out the remaining possibilities.

Type 2 partners and children experience diminished togetherness and negative changes to general feelings of closeness. Partners miss intimacy and sexuality. Partners feel lonely when carrying out activities on their own. It feels like a missed opportunity.

Where some of the type 2 caregivers adapt to the situation and report a proactive search for mutual interests like religion, music and grandchildren, other type 2 caregivers are unable to detach themselves from the situation. Although all type 2 caregivers seldom receive expressions of love, those caregivers who seem able to adapt talk about their efforts in recognizing signs of love, emotional closeness and companionship. They respect the care receiver and try to empathize with what it must be like for the person to have “this mental illness in everyday life”.

Those type 2 caregivers, who are unable to detach themselves from the situation, suffer with the suffering of the care-receiver. They feel lonely, show signs of weariness and feel lethargic. They even talk about a loss of respect for the care-receiver. They feel there is no open communication that allows them to enter each other’s world. After the many years of daily confrontation with clearly unchangeable behaviour they end up feeling victimized.

### The psychological well-being of the caregiver

The psychological well-being of informal caregivers encompasses the presence or absence of the following dimensions: grief and mourning, autonomy and meaning, and meaningful participation in social life.

#### Grief and mourning

All caregivers recognize the impact of the disease on the course of life for the care-receiver. There are differences in the way the two types experience grief and mourning.

Type 1 caregivers do not experience the difficult behaviour of the care receiver as intrusive to their daily life. They accept as a matter of fact that the care receiver needs practical support and they recognise the importance of regularly checking on the house and the condition of the care-receiver. They emphasize the importance of creating positive moments that they both can enjoy. Being able to establish a personal relationship with a person in need is felt to be rewarding, even if what one would like to accomplish is not achieved. Caregiving itself is considered to be an opportunity to give extra meaning to one’s life. These caregivers do not express feelings of grief and mourning. They can temporarily distance themselves from their responsibilities without feeling inadequate.

Type 2 caregivers grief about their incapacity to ease the care receiver’s suffering. Caregivers mourn for the disappearance of the healthy parent or partner. They talk about feelings of - sometimes complete - alienation in all aspects of the relationship and in their contacts with the social environment. They have experienced that participation in social events with their partner or parent is complicated because it is either too threatening for the care-receiver or too embarrassing for them as the care-receiver may behave in a way that is unpleasant. Caregivers feel sad about the loss of meaningful contact with important persons in their social environment. Together with the absence of reciprocity in social support, feelings of sadness and loneliness become part of the daily burden. This sorrow is exacerbated by their in-between position. They feel they have to mediate between the vulnerable person with often difficult behaviour, and other family members, the health care system, and financial agencies who do not really understand their troubles.

#### Autonomy and meaning

Autonomy is about the freedom or authority to govern one’s own life. It is about being a distinct person with a unique identity who has a purpose in life. The types differ in their experience of autonomy and meaning.

Type 1 caregivers experience caregiving as part of their chosen lifestyle and it represents a possibility for self-fulfilment. These caregivers balance their private time and their time spent on caregiving. If ever they exceed their time temporarily, they soon reduce it to what is considered an acceptable investment.

Type 2 caregivers’ autonomy changes over the years. Caregivers are confronted with the care receiver’s unchangeable patterns and rituals. They adapt their own activities to spare the care receiver, and to prevent further negative confrontations and difficult behaviour. The care-receiver takes precedence and some of caregivers feel they are forced to waive their own needs and desires.

In order to maintain caregiving, caregivers force themselves to be strong and therefore they have to cross psychological boundaries. They feel their autonomy is undermined by societal pressure to take responsibility for the care-receiver in controlling the consequences of the mental illness. The participating children report about their struggle to become an autonomous person after growing up with a mentally ill parent. All type 2 caregivers relinquished (part) of an anticipated lifestyle of their own. Some of these caregivers succeed in maintaining diminished but meaningful activities. They may try to overcome or compensate for their feelings of powerlessness and lack of recognition by getting involved in caregiving activities for other persons in need. This allows them to experience the reward and affirmation they miss. For instance, they may volunteer in a nursing home or look after their grandchildren. This gives them strength and confirms that the powerlessness and failure they otherwise experience is not their fault. Some of these caregivers have lost their identity. The mental illness rules their mind and their lives.

#### Meaningful participation in social life

Meaningful social participation is the extent to which the caregiver experiences a satisfying and constructive way of engaging in informal and formal relations.

The type 1 caregivers feel that caregiving adds something worthwhile to their life. They feel respected by others in their social environment for their efforts in caregiving. By serving the care receiver the caregiver is able to fulfil personal needs and values. They effectively put other sources of caregiving in place if they themselves are not able to meet care receiver needs.

The type 2 caregivers recognize the threat and danger of social exclusion. Because they manage everyday affairs by themselves, they often feel unable to change or improve their social environment. As a consequence they feel excluded from society. Some type 2 caregivers retain an often diminished participation in social life while others have no opportunities left for social participation, recreation or leisure. These type 2 caregivers often forget what activities they were formerly engaged in.

### Influential factors

The interviews further revealed other factors that may have a meaningful influence on the way the caregiving situation is perceived. These include resignation to the likelihood that there will be no improvement in the situation; sharing the same household; the question of feeling competent in dealing with the care-receiver's illness and his/her difficult behaviour; and the ability and/or willingness to ask for support from the social network.

Type 1 caregivers generally accept that the care recipient will not get better and that the situation will not improve. Since they do not share the same household with the care recipient, they have the opportunity to withdraw temporarily or even for a prolonged period from the care-receiver and his or her difficult behaviour. They feel competent in dealing with the situation because they are able to understand the problems and are creative in finding solutions. They can rely on their social network and ask for support. In this sense, these factors may act as resources to alleviate the impact of caregiving. Therefore, type 1 caregivers often describe caregiving as a process of gain.

In contrast, type 2 caregivers generally evaluate their situation as a process of loss. Due to their mutual dependency, the caregivers find it difficult to accept the care-receiver’s handicaps and (bleak) prognosis. When sharing the same household, caregivers are always confronted with the presence of the care-receiver and therefore do not have the possibility to withdraw from the caregiving situation and recover from the stress of caregiving, or find time to live their own life. As a consequence they may feel incompetent in dealing with the situation and because they do not want to bother other people with their problems they are reluctant to ask for support. These type 2 caregivers can feel entirely captive and may ultimately become harmful or aggressive towards the care-receiver. However, some type 2 caregivers remain feeling competent and do not hesitate to ask for support from the social network. This in return reinforces their feelings of competence. These type 2 caregivers have learned to adapt to the situation and have found balance in their life.

## Discussion

In this study the concept of freedom of choice is stated as the key concept in explaining the impact of long-term caregiving. With this concept of freedom of choice, a highly relevant new dimension is added to the understanding of the impact of caring on caregivers’ wellbeing. Perceived freedom of choice shows that differences in impact cannot be explained solely on the basis of stressors, buffers and contextual factors found in the research to date. Freedom of choice appears to give coherence to the factors that aggravate caregiving. This adds a new perspective to the research on caregiver burden. In order to ensure that the concept of freedom of choice is not itself a result of the burden process, the first (M.I.Z) and the third (M.G.) authors reread several of the interviews. The interviews were chosen at random and the researchers focussed on looking for confirmation of this possible reversal. It appears that reversal does not match the stories of the participants.

Thinking in proto types helped us to uncover this differentiating concept of freedom of choice. Perceived freedom of choice underpins our definition of the two main types of caregiver. For those who have a perceived freedom of choice to engage in caregiving – the type 1 caregivers - caregiving is mainly a process of gain, despite the invested time and energy. Caregiving gives extra meaning to their lives. For those who do not perceive they have the freedom to quit caregiving - the type 2 caregivers - caregiving is experienced as a process of loss. More importantly than the time and energy invested in caregiving tasks, for the type 2 caregivers it is the experience of the virtual loss of their partner or parent that makes their situation difficult to bear.

Within this group of type 2 caregivers we can identify two subtypes. The first subtype is the caregiver who accepts the loss and caregiving as part of their life and of which they have to bear the consequences. They manage to adapt their expectations. They are able to notice reciprocity and they experience togetherness by interpreting reciprocity. To some extent they retain autonomy while they reflect on a poorer but nonetheless meaningful life. The second subtype is the caregiver who feels captured. On one hand they cannot imagine a life without the care-receiver, while on the other hand they suffer because their sick partner or parent never shows any signs of gratitude. They do not consent to the consequences but can also not evade them. These caregivers feel absorbed by the demands and cannot handle caregiving.

This study has shown that the loss is mainly felt within the quality of the relationship and in psychosocial wellbeing. In fact, all levels of interaction between caregivers, care-receivers, their social environment, and the interpersonal relationship are affected by caregiving. Relationships become unequal; frail relationships and caregivers’ psychosocial wellbeing are negatively affected. This study points out that, besides attention to determinants of burden [5,7,10,11,14,18,20,22] and reducing the tasks of the care receiver, more attention should be given to the consequences for the type 2 caregivers’ individual emotional needs and well-being. The results of this study are in line with existing, though limited, research findings on caregiver burden. These include lack of self-actualization [29], the importance of reciprocal social relationships [30], changed meaning in life, the profound sense of loss of companionship and intimacy, fulfilment of family roles, adjustment to persistent grief, as well as major disruptions to expectations for the future [1,21,23,24,31]. As the care-receiver and caregiver are together on a daily basis, the impact of caregiving is not caused by a single event, but by a series of events that confirm the sense of the loss, in the context of long-term disability. The concept of freedom of choice might add to our understanding of the differences outlined above and explain the variation in impact on the caregivers’ life. The concept of different types of caregiver is acknowledged in studies by [32,33].

For professional practice this recognition of freedom of choice is important. In the assessment of caregiver needs the MHCN ought to assess this freedom of choice as part of the assessment. Type 2 caregivers are at risk and are therefore in need of support. It is likely that type 1 caregiver’s wellbeing is not as much at risk as the wellbeing of the subtypes within the second group. Therefore support for type 1 caregivers might be mainly practical and appreciative. Our findings not only point the need for practical advice and information about illness and illness-related issues but also to a further differentiation in a narrative coaching on themes such as loss and grief and how to find new meaning. Hence, support programs should not only be designed to reduce the symptoms of burden, but should focus on the wellbeing of caregivers and apply a competence paradigm for professional practice [34]. An intervention should ideally use an empowering approach to encourage caregivers to redefine their personal life and focus on their caregiving strengths, and on 'living’, rather than on the reduction of caregiving tasks and their subjective burden. A relationship review could form part of the intervention. As suggested by other research, a pro-active approach is advised because the caregiver is often no longer able to ask for help. Regarding the nature of chronic mental illness and the differences in caregiver types, support programs for type 2 caregivers should consist of complex, multivariate interventions that are comprehensive, long-term, individually tailored and have the flexibility to meet the dynamics of caregiving over time.

The concept of freedom of choice may have implications for stress-coping theory as elaborated in the theory of Lazarus and Folkman [9]. Although freedom of choice is not a concept the caregiver is aware of, it influences the caregiver's appraisal of stressors. The extent to which the caregiver experiences the possibility to end the confrontation with main stressors like care, difficult behaviour or emotional consequences might influence their primary coping.

### Strength’s and limitations

#### Strengths

To maximize our knowledge about the appraisal of the caregiving situation, we sought sampling diversity for type of relationship, gender, type of illness and institute, as these characteristics may influence the way the caregiver experiences the situation. This diversity provided different perspectives on the appraisal of long-term caregiving. These different perspectives facilitated constant comparison between cases. We conclude that we gathered rich data that sustained our insight into the process of appraisal.

To avoid ‘going native’ [35] and the development of blind spots prejudicing the complete process, in addition to researcher triangulation the analyses were discussed with a third researcher (M.J.A.) with a different disciplinary background.

#### Limitations

Despite sampling diversity we realize that we included only those caregivers who maintained caregiving. Although we have substantial variation in the perspectives of the participants, including participants who stopped caregiving could possibly have deepened our analysis. The respondents within this convenience sample were approached by their mental health care nurse. A total of twenty four caregivers were invited to join the study. Largely for emotional reasons, five caregivers declined to participate. During the interviews the participants were able to share emotional feelings about the care they provided and the impact of caregiving on their lives. We therefore think that it was still possible to gain insight into their emotions.

## Conclusions

The concept of freedom of choice adds to our understanding of the differences and explains the variation in impact on the caregivers’ life. The type 1 caregiver generally experiences gain whereas type 2 generally experiences loss, which puts the latter group typically at risk of becoming overloaded. Whether people perceive that they have freedom of choice in caregiving is an important consideration in evaluating the type of intervention needed to support caregivers.

## Competing interests

The authors declare that they have no competing interests.

## Authors’ contributions

MIZ was leader of the conceptualization and design of the study, data collection, analysis and drafting and revising the manuscript. MJA participated in the data collection, the analysis of the qualitative data and the drafting and revision of the manuscript. MG contributed to the conceptualization and design of the study, conducted analyses and reporting on the qualitative data, reviewed qualitative findings and revision of the manuscript. PC contributed to the conceptualization and design of the study and the interpretation of qualitative data, and the drafting and revising of the manuscript. All authors read and approved the final manuscript.

## Pre-publication history

The pre-publication history for this paper can be accessed here:

http://www.biomedcentral.com/1471-244X/13/103/prepub

## Supplementary Material

Additional file 1 Topic list of semi-structured interviews. Click here for file
