# Supplementary material for: Differences in impact of long term caregiving for mentally ill older adults on the daily life of informal caregivers: a qualitative study
Source: BMC Psychiatry. 2013 Mar 27;13:103. doi: 10.1186/1471-244X-13-103 (PMC3617010; doi:10.1186/1471-244X-13-103)
Supplement: Additional file 1 — Topic list of semi-structured interviews. [file 1471-244X-13-103-S1.docx]

Additional file 1 Topic list semi-structured interviews.

| Caregivers' story of the illness and how it started |
| --- |
| Elements of the caregiving role   - tasks - responsibilities - day-to-day management |
| Caregivers’ thoughts, feelings, and responses related to interaction with the care receiver |
| Caregivers’ thoughts, feelings, and responses related to conversations with the care receiver |
| Changes in the course of their own life |
| Caregiver’s own emotions, grief and losses |
| The impact on the quality of the relationship |
| Living with difficult behaviour |
| Thoughts about the future |
